# A Comparative Analysis of Microscopy, Culture, and the Xpert Mycobacterium tuberculosis/Rifampicin Assay in Diagnosing Pulmonary Tuberculosis in Human Immunodeficiency-Positive Individuals

**DOI:** 10.7759/cureus.42962

**Published:** 2023-08-04

**Authors:** Manish Kumar, Gudesh Kumar, Rakesh Kumar, Sweta Muni, Satyadeo Choubey, Shailesh Kumar, Namrata Kumari

**Affiliations:** 1 Microbiology, Indira Gandhi Institute of Medical Sciences, Patna, IND; 2 Pulmonary Medicine, Indira Gandhi Institute of Medical Sciences, Patna, IND

**Keywords:** xpert mtb/rif assay, pulmonary tuberculosis, hiv-infected patients, diagnosis, culture

## Abstract

Introduction: Individuals with human immunodeficiency virus (HIV) infection have an increased likelihood of developing tuberculosis (TB). The primary objective of this study was to compare the diagnostic accuracy of microscopy, culture, and the Xpert *Mycobacterium tuberculosis*/Rifampicin (MTB/RIF) assay in the diagnosis of pulmonary TB in sputum samples of HIV-infected patients. The secondary objectives were to evaluate the sensitivity and specificity of these three methods along with a comparison of diagnostic approaches for detecting drug-resistant strains.

Material and methods: This prospective, laboratory-based study was done in the Microbiology Department of IGIMS, Patna. The study included sputum samples of 102 individuals who were HIV-positive and exhibited symptoms indicative of tuberculosis.

Results: Out of 102 individuals suspected of having tuberculosis, 18 tested positive for *M. tuberculosis*. Male individuals between the ages of 31 and 40 were more affected by both HIV and tuberculosis, and in most of these cases, their CD4 cell count was below 200 cells/µl. Among the 102 sputum samples collected, 18% (18 samples) were found to be positive using the Mycobacterium Growth Indicator Tube (MGIT) 960 liquid culture method. Two samples were contaminated, and 14.7% (15 cases) tested positive using the cartridge-based nucleic acid amplification test (CBNAAT) method. Additionally, 3.92% (four samples) were positive using the ZN staining method.

Conclusion: The study found that Xpert MTB/RIF outperformed other methods in identifying resistance to RIF, showed better agreement with gene sequencing results for RIF resistance, and had higher accuracy in detecting tuberculosis cases, including both smear-positive and smear-negative cases.

## Introduction

More than 200 countries and regions, which comprise almost all the population of the world, have outlined data pertaining to tuberculosis (TB) cases in 2022. The various diagnostic aids for TB control are either old or have become ineffective and may not be reachable in many places, and pulmonary TB diagnosis is mostly based on sputum examination. A novel methodology is the need of the hour to diagnose TB and help eliminate this serious public issue. The amalgamation of human immunodeficiency virus (HIV) and TB forms a deadly combination and hastens each other’s progression. Around 1,87,000 deaths have been reported in 2021 in cases of HIV-associated TB. There has also been an increase of 3% in cases of HIV in TB from 2020 to 2021. The maximum burden of HIV-associated TB is present in the African sub-continent [1,2]. The World Health Organization has continuously stressed the fact that TB remains a substantial public health issue among poor countries and developing nations [3]. The loss of CD4+ T cells in the blood, lymphatics, and mucous membrane is the main characteristic feature in HIV-infected individuals and contributes to the risk of TB, which occurs early when CD4 T cells are in the normal range [4]. HIV promotes these epidemics of tuberculosis in several ways, like accelerating advancement to active tuberculosis and latent tuberculosis reactivation [5]. Given the intricate nature of diagnosing and treating both TB and HIV/AIDS concurrently, it is imperative that significant attention be given to this issue [6]. In addition to imposing a significant health burden across the globe, TB stands as the 10th major contributor to global mortality. Surpassing HIV/AIDS, it emerges as a prominent cause of death resulting from infectious diseases [7]. The primary objective of the End TB Strategy (2016-2035) by WHO is to eradicate the tuberculosis epidemics worldwide by 2035. This strategy encompasses specific goals such as decreasing TB mortality by 95% and reducing incidence by 90% within the period from 2015 to 2035. Additionally, it aims to guarantee that families affected by TB will no longer endure catastrophic financial burdens related to the disease by the year 2030 [8]. Individuals with HIV infection face a significantly elevated relative risk, approximately 20 to 30 times greater, of developing tuberculosis throughout their lifetimes [9]. The focus is to prioritize early and dependable diagnosis for every individual with TB, ensure appropriate medications, and establish comprehensive patient care support systems that encompass financial aid and nutritional assistance. Furthermore, preventive measures like TB vaccines and TB preventive treatment [10] enhance this strategy. The major hurdle to achieving tuberculosis control is the challenge of achieving a precise disease diagnosis [11]. When TB diagnosis is delayed, not only does the risk of mortality increase, but there are also increased chances of a hyperinflammatory response in those undergoing antiretroviral therapy [12].

The study was also done to ascertain the prevalence of pulmonary tuberculosis among individuals who are HIV-positive and seeking medical care at IGIMS in Patna. The main objective of this research was to assess the reliability of microscopy, culture, and the Xpert *Mycobacterium tuberculosis*/rifampicin (MTB/RIF) assay in detecting pulmonary tuberculosis (TB) in sputum samples from individuals with HIV infection. The secondary objectives of the study involved assessing the sensitivity, specificity, and predictive values of the three methods along with potential relationships between the CD4+ T-cell count and HIV viral load.

## Materials and methods

This laboratory-based prospective and comparative study was done in the Microbiology Department, IGIMS, Patna. The inclusion criteria were patients of age 18 and above with cardinal manifestations indicative of TB like a persistent cough, pyrexia, loss of weight, and sweats at night who had attended the Integrated Counselling and Testing Centre (ICTC) or pulmonary medicine department and were investigated in the Department of Microbiology at IGIMS Patna. Tests like Xpert MTB/RIF, culture, and microscopy of sputum were performed on all individuals with HIV who tested positive for the virus to determine the presence of *M. tuberculosis*. The study was performed between December 2020 and June 2022. One hundred and two patients who met the above criteria were selected. Prior to their participation, the patients were informed about the experimental design and provided with written informed consent. Additionally, a comprehensive clinical history was recorded using a prepared Proforma. The research protocol received approval from the Institutional Ethical Committee of IGIMS, Patna (1962/IEC/IGIMS/2020).

Method of collection of specimens

The patients were provided with counselling regarding the importance of obtaining a high-quality sample and the steps to follow in order to avoid contamination of the sample and collection container. Two wide-mouthed containers were given to each patient, and they were instructed to expectorate after a deep breath without mixing saliva. The volume of the specimen collected was between 3 and 5 ml, either mucoid or mucopurulent in nature. The patients were requested to provide two samples: one spot sample and another in the early morning. The processing of the spot sample involved both Mycobacterium Growth Indicator Tube (MGIT) and GeneXpert tests, as well as microscopy, while the morning sample collected on the following day was processed only for microscopy. The staining methods used for microscopy followed the direction of the Revised National Tuberculosis Control Programme (RNTCP) and utilized Ziehl-Neelsen (ZN) staining.

The sputum specimens underwent processing using the Xpert MTB/RIF assay, following the guidelines provided by the Central TB Division of the Government of India (RNTCP, 2013; RNTCP, 2012). The assay involved the extraction and amplification of MTB-Complex DNA and the rpoB gene to detect rifampicin (RIF) resistance. To achieve a high level of specificity, specific primers (3) and five molecular beacons were utilized (WHO, 2014). The interpretation of the results was reliant on the existence or non-existence of MTB, as well as the detection or non-detection of RIF resistance. In cases where the determination of RIF resistance was inconclusive or invalid, the positive beacon played a crucial role. Samples of sputum were treated with a sodium hydroxide and isopropanol solution, including a reagent sample (SR). Then the incubation was done at ambient temperature for 15 minutes. The sample under treatment was conveyed to a cartridge and inserted into the GeneXpert system. The following steps for processing were completely automated.

To perform liquid culture, a volume of 0.5 ml from a properly mixed, processed, and concentrated sputum specimen was inoculated onto a liquid medium called BD BACTECTM MGITTM 960 (BD BACTEC, Rutherford, NJ, USA). The culture was then examined (BD BACTEC), following the instructions provided by the manufacturer. If a culture tested positive, it underwent subculture on a blood agar plate, Ziehl-Neelsen (ZN) staining, and TBcID (an immunochromatographic test) for further confirmation of detecting *M. tuberculosis* complex.

Statistical analysis

The information in question was organized in a Microsoft Excel spreadsheet (Microsoft® Corp., Redmond, WA) and examined for any connections or relationships. Statistical data were analyzed with SPSS software (version 22, IBM Corp., Armonk, NY, USA). To determine various measures, such as sensitivity, specificity, positive and negative predictive value (PPV and NPV), and accuracy, the culture of *M. tuberculosis* from a sample was used as the benchmark or gold standard. True positives and true negatives were identified based on whether they tested positive or negative in the culture method, respectively. False positive samples were those that came in culture-negative but positive on the GeneXpert test, while false negative samples were those that came in culture-positive but negative on the GeneXpert test, as well as in the ZN smear test.

## Results

**Figure 1:**
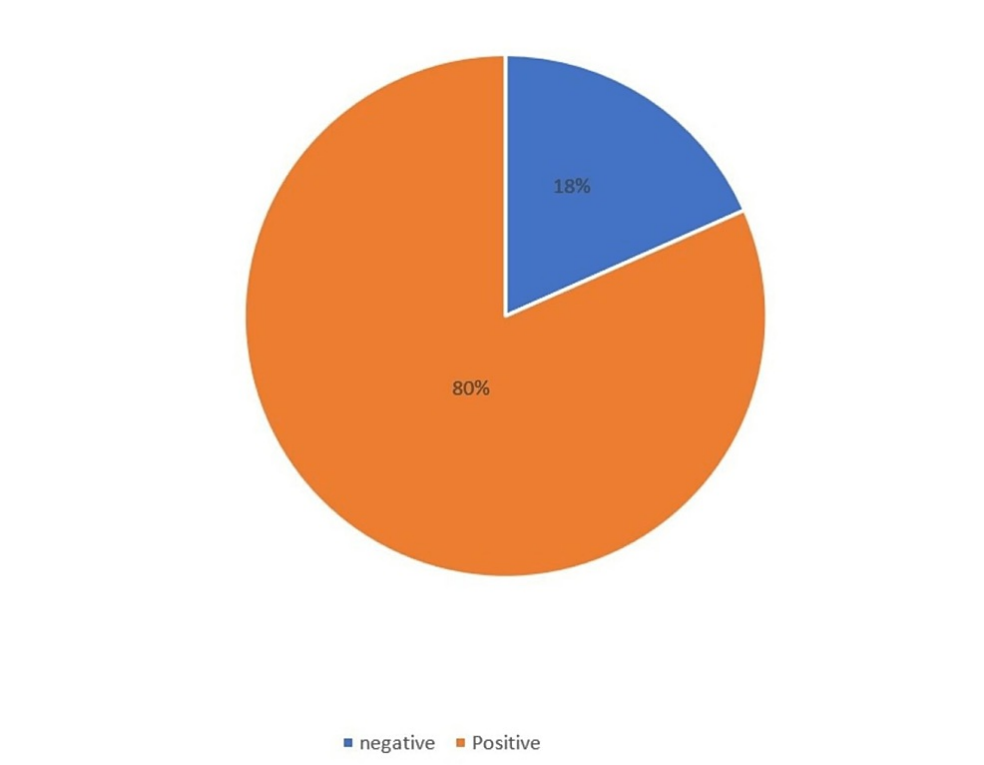
Findings of Mycobacterium tuberculosis in HIV-positive individuals

**Figure 2:**
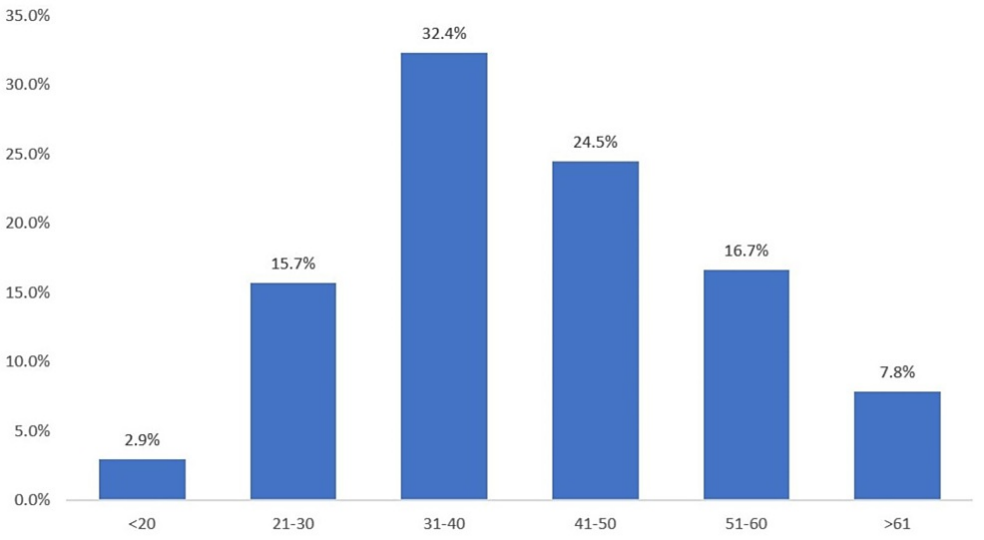
Age distribution analysis of the entire cases

Among the 102 sputum samples that were examined, *M. tuberculosis* was found to be positive in 18 cases, accounting for 17.64% of the total samples (Figure 1). There was a higher occurrence of males, constituting 76%, and a substantial portion of suspected cases fell within the 31-40 age range, comprising 32.4% (Figure 2). The most common symptoms observed among our study group were fever (80.4%), cough (62.7%), and weight loss (53.9%), followed by breathlessness (7.8%) and hemoptysis (6.9%) (Table 1). It should be noted that CD4 count testing was only possible for 51 cases. In most of these cases, the CD4 count was < 100 cells/μl (12.7%), followed by between 100-200 cells/μl (10.8%) and 301-400 cells/μl (8.8%) (Table 2). The CD4 count distribution of AFB smear-positive specimens was <200 cells/μl in 100% of cases, Xpert-positive CD4 count was <200 cells/μl in 83.3% of cases, and for MGIT-positive culture, CD4 count was <200 cells/μl in 85.5% of cases. This reflects the fact that patients with low CD4 counts are more susceptible to pulmonary tuberculosis (Table 3).

**Table 1:** CD4 count cells among age groups

CD4 count	Frequency	Percent
<100	13	12.7%
101–200	11	10.8%
201–300	3	2.9%
301–400	9	8.8%
401–500	7	6.9%
501–600	4	3.9%
>601	3	2.9%
NA	52	51.0%
Total	102	100.0%

**Table 2:** Analysis of symptom distribution among the cases

Complaints	Frequency	Percent
Cough	64	62.7%
Breathlessness	8	7.8%
Fever	82	80.4%
Weight loss	55	53.9%
Hemoptysis	7	6.9%
Decreased appetite	28	27.5%

**Table 3:** Statistical analysis of results by various methods with CD4 count number

Methods	CD4 counts	<100	101–200	201–300	301–400	401–500	501–600	>601	NA
ZN Staining	Negative	11	9	3	9	7	4	3	52
	Positive	2	2	0	0	0	0	0	0
CB NAAT	Negative	10	4	2	8	7	4	3	49
	Positive	3	7	1	1	0	0	0	3
MIGIT	Negative	8	3	2	8	7	4	3	49
	Positive	5	8	1	1	0	0	0	3

**Figure 3:**
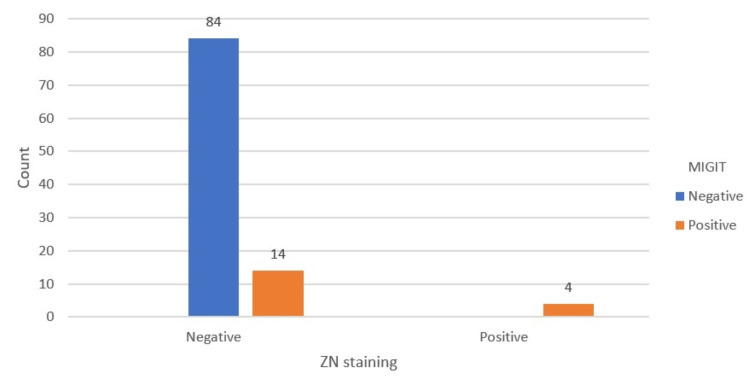
Comparison of ZN stain results with culture (considered as the gold standard) depicted in a bar chart

A collective of 102 sputum samples underwent MGIT 960 culture testing. Two sputum samples tested with MGIT 960 were deemed "contaminated" and were excluded from this analysis. Out of the other 100 samples, 18% (18 out of 100) tested positive, while 80% (82 out of 100) tested negative. AFB smears showed a positive result in 3.92% (4 out of 102) of cases. The sensitivity of the AFB smear, when compared to the MGIT culture, was 22.22%, and the specificity was 100%. The PPV was 100%, and the NPV was 85.71%. In summary, the accuracy of the AFB smear test was 86.27% (Figure 3 and Table 4).

**Table 4:** Performance metrics (sensitivity, specificity, PPV, NPV, and accuracy) of ZN stain compared to culture

Parameter	Estimate (%)
Sensitivity	22.22%
Specificity	100.00%
PPV	100.00%
NPV	85.71%
Accuracy	86.27%

**Figure 4:**
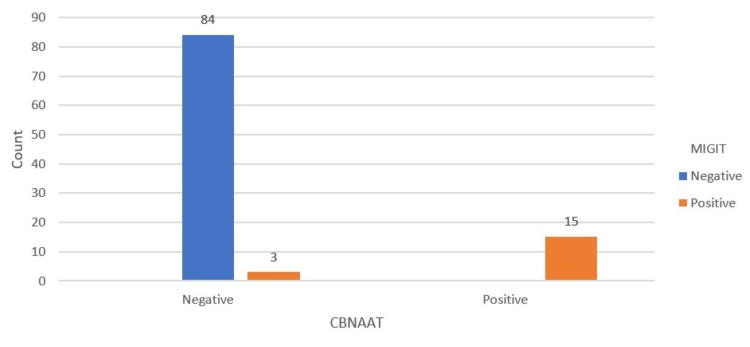
Bar chart showing comparison of results of CBNAAT with culture (gold standard) CBNAAT: cartridge-based nucleic acid amplification test

**Figure 5:**
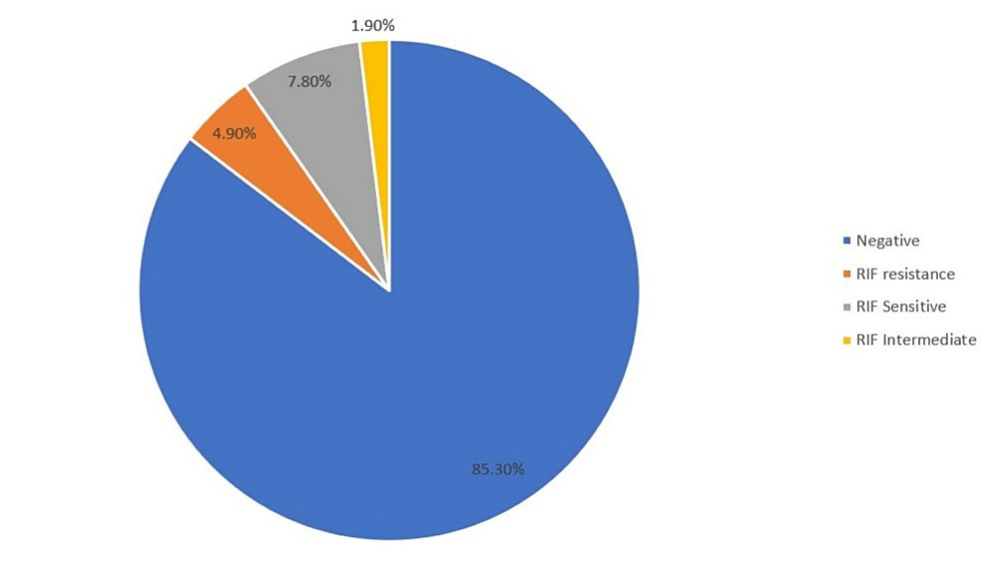
Distribution of rifampicin susceptibility revealed by the Xpert MTB/RIF assay depicted in a pie chart

Out of 102 cases, the GeneXpert testing produced a positive result in 14.7% (15 cases). The results of this assay demonstrated an elevated sensitivity of 83.3% and a specificity of 100%. The PPV was 100%, and the NPV was 96.55%. The total accuracy of the GeneXpert assay was 97.06% (Figure 4 and Table 5). In addition, the GeneXpert assay identified rifampicin resistance in 4.9% (5 cases out of 102) (Figure 5). Among HIV patients, the highest association was found with anemia (8.8%), followed by chronic kidney disease (3.9%), thrombocytopenia (2.9%), hepatitis B, and one case of chronic liver disease (1%).

**Table 5:** Sensitivity, specificity, PPV, NPV and accuracy value of CBNAAT with culture (gold standard) PPV: positive predictive value; NPV: negative predictive value; CBNAAT: cartridge-based nucleic acid amplification test

Parameter	Estimate (%)
Sensitivity	83.33%
Specificity	100.00%
PPV	100.00%
NPV	96.55%
Accuracy	97.06%

## Discussion

The available data on the performance of GeneXpert testing in HIV-affected individuals is currently limited, and there is a paucity of specific data categorizing them according to CD4 count [13]. The purpose of this study was to assess the predictive values and diagnostic accuracy of the microscopic method compared to culture and the CBNAAT (Cartridge-Based Nucleic Acid Amplification Test) in individuals with HIV and TB co-infection.

In this study, most patients were male (73.5%), which aligns with previous research conducted by Geleta et al. and Arora et al. [14,15]. The increased number of males in this study can be linked to their migratory patterns, which expose them to unemployment and high-risk activities. Conversely, a considerable proportion of females within the population are uneducated and primarily occupied with domestic responsibilities, resulting in limited engagement with routine healthcare services. Consequently, this tendency often leads to underreporting of their health status. The most common symptom in our study was fever among positive cases, which is comparable with Affusim et al. and Cain et al. [16,17].

Furthermore, among the 18 individuals who tested positive for tuberculosis, CD4 cell count assessments were conducted in 15 cases. The analysis revealed that 23.5% of the patients exhibited a CD4 cell count lower than 200 cells/μl, with 12.7% falling below 100 cells/μl and 10.8% ranging from 101 to 200 cells/μl. The findings align with earlier investigations carried out by different researchers [18,19].

In relation to Z-N staining, our study observed that the sensitivity, specificity, PPV, NPV, and accuracy were 22.2%, 100%, 100%, 85.71%, and 86.27%, respectively, when compared to culture as the reference standard. These outcomes align with the findings reported by Pierrae et al. and Agrawal et al. [20,21].

Within our research, the CBNAAT demonstrated a sensitivity of 83.3%, a specificity of 100%, a PPV of 100%, a NPV of 96.5%, and an accuracy of 97.06%. These findings are consistent with other research, including the analysis undertaken by Pierrae et al. [20], Mukako et al. [22], and Coung et al. [23], which reported sensitivities of 80%, 74.9%, and 66.7%, respectively. The observed lower NPV in our study can be attributed to two samples that were culture-positive but tested negative with the GeneXpert assay. The possible reasons for this observation might be attributed to a decreased amount of bacteria present, which makes it challenging for GeneXpert to identify the MTB DNA, or the presence of substances that hinder the PCR amplification enzyme in the test sample. These findings indicate that individuals who receive a negative GeneXpert result may still be infected with tuberculosis. Furthermore, our study revealed a RIF resistance prevalence of 38.46%, which is lower than the rates reported in research conducted by Ganguly et al. [24] and Arora et al. [15]. This difference could be attributed to variations in the mutations responsible for drug resistance across different geographical regions.

Despite being acknowledged as an excellent molecular diagnostic test for tuberculosis, the Xpert MTB/RIF assay has some drawbacks. A significant drawback is that it depends on identifying resistance detected in rifampicin (RIF) for indicating or classifying a sample as having multidrug-resistant TB (MDR-TB). However, certain strains that may be resistant only to rifampicin might not require the full course of MDR therapy. This can lead to an overestimation of the number of MDR-TB patients [25]. Likewise, research conducted in Mumbai, India, revealed that samples categorized as rifampicin-sensitive based on GeneXpert testing could potentially exhibit resistance to isoniazid [26]. The Xpert MTB/RIF assay presents additional limitations that should be taken into consideration. These include the need for a dependable power supply, strict temperature control, and regular instrument calibration on an annual basis. Hence, these technical requirements should be carefully addressed and managed to ensure the accurate and reliable execution of the gene Xpert testing. Despite these limitations, the Xpert assay has been adopted as a TB diagnostic method due to its clear results, speed, and high sensitivity.

Limitations

Clinical follow-up and a reference standard for the samples analyzed by Xpert MTB/RIF and culture were not provided, as there was no ongoing monitoring of the patients. Additionally, the study had a comparatively limited sample size collected from only one healthcare facility, limiting the generalizability of the findings to the entire population. Therefore, further studies involving larger and more diverse populations from different regions of the country are necessary to establish definitive conclusions. Nevertheless, the primary objective of our study, which was to assess the efficacy of the Xpert assay in tuberculosis specimens, was effectively accomplished compared to microscopy and MGIT culture, focusing on clinically suspected TB cases in HIV patients.

## Conclusions

The Xpert MTB/RIF assay demonstrates outstanding performance in detecting rifampicin resistance and shows substantial accordance with gene sequencing outcomes for detecting resistance to rifampicin. Additionally, it exhibits higher sensitivity and specificity in diagnosing both positive and negative smears in cases of tuberculosis. Xpert MTB/RIF offers a practical solution in areas with limited resources and inaccessible locations where it is challenging to set up a laboratory that complies with the required biosafety criteria. The widespread implementation of this assay has the probability of notably increasing the rates of detecting drug-sensitive strains and MDR-TB, leading to timely management decisions and ultimately reducing disease transmission.

## References

[1] (2023). Global tuberculosis report 2022. https://www.who.int/publications/i/item/9789240061729.

[2] Lienhardt C, Espinal M, Pai M, Maher D, Raviglione MC (2011). What research is needed to stop TB? Introducing the TB research movement. PLoS Med.

[3] Payen MC, VA Vooren JP, Vandenberg O, Clumeck N, DE Wit S (2017). Isolation unit for multidrug-resistant tuberculosis patients in a low endemic country, a step towards the World Health Organization End TB Strategy. Epidemiol Infect.

[4] Bruchfeld J, Correia-Neves M, Källenius G (2015). Tuberculosis and HIV coinfection. Cold Spring Harb Perspect Med.

[5] Lönnroth K, Castro KG, Chakaya JM, Chauhan LS, Floyd K, Glaziou P, Raviglione MC (2010). Tuberculosis control and elimination 2010-50: cure, care, and social development. Lancet.

[6] Gesesew H, Tsehaineh B, Massa D, Tesfay A, Kahsay H, Mwanri L (2016). The prevalence and associated factors for delayed presentation for HIV care among tuberculosis/HIV co-infected patients in Southwest Ethiopia: a retrospective observational cohort. Infect Dis Poverty.

[7] Bajrami R, Mulliqi G, Kurti A, Lila G, Raka L (2016). Comparison of GeneXpert MTB/RIF and conventional methods for the diagnosis of tuberculosis in Kosovo. J Infect Dev Ctries.

[8] Raviglione MC, Uplekar MW (2006). WHO's new Stop TB strategy. Lancet.

[9] Manosuthi W, Chottanapand S, Thongyen S, Chaovavanich A, Sungkanuparph S (2006). Survival rate and risk factors of mortality among HIV/tuberculosis-coinfected patients with and without antiretroviral therapy. J Acquir Immune Defic Syndr.

[10] (2023). Coming together to end TB altogether. https://tbcindia.gov.in/WriteReadData/IndiaTBReport2022/TBAnnaulReport2022.pdf.

[11] Young DB, Perkins MD, Duncan K, Barry CE 3rd (2008). Confronting the scientific obstacles to global control of tuberculosis. J Clin Invest.

[12] Balcha TT, Sturegård E, Winqvist N (2014). Intensified tuberculosis case-finding in HIV-positive adults managed at Ethiopian health centers: diagnostic yield of Xpert MTB/RIF compared with smear microscopy and liquid culture. PLoS One.

[13] Theron G, Peter J, van Zyl-Smit R (2011). Evaluation of the Xpert MTB/RIF assay for the diagnosis of pulmonary tuberculosis in a high HIV prevalence setting. Am J Respir Crit Care Med.

[14] Geleta DA, Megerssa YC, Gudeta AN, Akalu GT, Debele MT, Tulu KD (2015). Xpert MTB/RIF assay for diagnosis of pulmonary tuberculosis in sputum specimens in remote health care facility. BMC Microbiol.

[15] Arora D, Jindal N, Bansal R, Arora S (2015). Rapid detection of Mycobacterium tuberculosis in sputum samples by Cepheid Xpert assay: a clinical study. J Clin Diagn Res.

[16] Affusim CC, Kesieme E, Abah VO (2012). The pattern of presentation and prevalence of tuberculosis in HIV-seropositive patients seen at Benin City, Nigeria. Int Sch Res Notices.

[17] Cain KP, McCarthy KD, Heilig CM (2010). An algorithm for tuberculosis screening and diagnosis in people with HIV. N Engl J Med.

[18] Swarooprani N, Wadekar MD, Rajakumar S (2016). Impact of CD4 count on sputum smear for AFB in HIV-TB Co infection. Indian J Microbiol Res.

[19] Yendewa GA, Lakoh S, Jiba DF (2022). Hepatitis B virus and tuberculosis are associated with increased noncommunicable disease risk among treatment-naïve people with HIV: opportunities for prevention, early detection and management of comorbidities in Sierra Leone. J Clin Med.

[20] Le Palud P, Cattoir V, Malbruny B (2014). Retrospective observational study of diagnostic accuracy of the Xpert® MTB/RIF assay on fiberoptic bronchoscopy sampling for early diagnosis of smear-negative or sputum-scarce patients with suspected tuberculosis. BMC Pulm Med.

[21] Agrawal M, Bajaj A, Bhatia V, Dutt S (2016). Comparative study of GeneXpert with Zn stain and culture in samples of suspected pulmonary tuberculosis. J Clin Diagn Res.

[22] Mukoka M, Twabi HH, Msefula C (2023). Utility of Xpert MTB/RIF Ultra and digital chest radiography for the diagnosis and treatment of TB in people living with HIV: a randomised controlled trial (XACT-TB). Trans R Soc Trop Med Hyg.

[23] Cuong NK, Ngoc NB, Hoa NB, Dat VQ, Nhung NV (2021). GeneXpert on patients with human immunodeficiency virus and smear-negative pulmonary tuberculosis. PLoS One.

[24] Ganguly J, Ray S, Nandi S, Halder S, Kundu S, Mandal A (2015). A study to evaluate pattern of rifampicin resistance in cases of sputum positive pulmonary tuberculosis. J Evol Med Dent Sci.

[25] Sharma SK, Kohli M, Yadav RN (2015). Evaluating the diagnostic accuracy of Xpert MTB/RIF assay in pulmonary tuberculosis. PLoS One.

[26] Vadwai V, Boehme C, Nabeta P, Shetty A, Rodrigues C (2012). Need to confirm isoniazid susceptibility in Xpert MTB/RIF rifampin susceptible cases. Indian J Med Res.

